# Electric Potential at the Interface of Membraneless Organelles Gauged by Graphene

**DOI:** 10.1021/acs.nanolett.3c02915

**Published:** 2023-10-20

**Authors:** Christian Hoffmann, Gennadiy Murastov, Johannes Vincent Tromm, Jean-Baptiste Moog, Muhammad Awais Aslam, Aleksandar Matkovic, Dragomir Milovanovic

**Affiliations:** † Laboratory of Molecular Neuroscience, German Center for Neurodegenerative Diseases (DZNE), 10117 Berlin, Germany; ‡ Chair of Physics, Department Physics, Mechanics and Electrical Engineering, Montanuniversität Leoben, 8700 Leoben, Austria

**Keywords:** biomolecular condensates, membranes, graphene, charge transfer, synapse

## Abstract

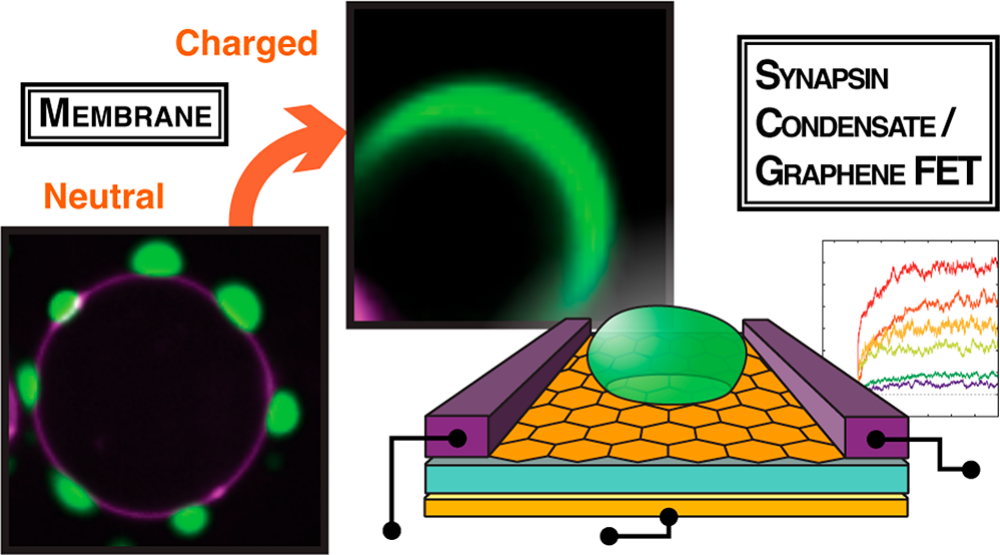

Eukaryotic cells contain membrane-bound and membrane-less organelles that are often in contact with each other. How the interface properties of membrane-less organelles regulate their interactions with membranes remains challenging to assess. Here, we employ graphene-based sensors to investigate the electrostatic properties of synapsin 1, a major synaptic phosphoprotein, either in a single phase or after undergoing phase separation to form synapsin condensates. Using these graphene-based sensors, we discover that synapsin condensates generate strong electrical responses that are otherwise absent when synapsin is present as a single phase. By introducing atomically thin dielectric barriers, we show that the electrical response originates in an electric double layer whose formation governs the interaction between synapsin condensates and graphene. Our data indicate that the interface properties of the same protein are substantially different when the protein is in a single phase versus within a biomolecular condensate, unraveling that condensates can harbor ion potential differences at their interface.

A cell is a complex concoction composed of many biomolecules, ions, and small metabolites, many of which are charged under physiological pH. Despite chemical complexity, a cell is well organized in membrane-bound compartments. Additionally, macromolecules demix from surrounding cyto-/nucleoplasm in membrane-less organelles, also known as biomolecular condensates.^1^ These two modes of compartmentalization are functionally coupled to each other. For instance, membranes can locally concentrate macromolecules thereby lowering the threshold concentration necessary for their condensation.^2,3^ Moreover, recent data show that condensates exert capillary forces on membranes yielding to their remodeling.^4,5^ A recent computational study characterized the differences among the inside, the outside, and the interface of condensates, indicating that the interface of condensates has distinct conformational characteristics.^6^ Here, we investigate the unique properties of interfaces by asking if the distinct conformational characteristics contribute to setting up an Electric Double Layer (EDL). This is further motivated by recent data indicating that the interfacial fields can drive spontaneous redox reactions.^7^ Thus, the interface of biomolecular condensates plays a critical role in regulating cell signaling and intracellular trafficking. However, the electrical properties at the interface of condensates remain largely elusive. This is due to the lack of techniques to directly measure the electric field gradient at the interface of biomolecular condensates and membranes.^8^

Inspired by the exceptionally sensitive field modulation response of graphene,^9^ electric potential and charge transfer gauges based on graphene field effect transistors (Gr FETs) and other two-dimensional (2D) materials have been utilized to probe the interaction with small organic molecules.^10−12^ In biosensing, classically optical and plasmonic-based sensors are used to detect biomolecules and track their transitions and charged states.^13,14^ A recent example includes plasmonic-based sensors of vitamin B12 that rely on the strong light-matter coupling of graphene nanoholes and nanoribbons.^15^ However, these are all indirect measures of electrical activity, whereas a pure electrical readout would allow for enhanced sensitivity and the reduction of the sensor complexity, size, and costs. To achieve sufficient sensitivity and desired selectivity many different nanostructured materials have been utilized in bioelectronics.^16^ Especially, 2D material-based sensors are well-suited to probe interfacial electric fields and charge transfer due to their capability of fast readouts and exceptional sensitivity.^17,18^ Specifically, Gr FETs can be used label-free because their sensing capabilities depend on the measurement of the changes in electrical properties of the device active area upon interaction with an analyte.^19^

Due to all these unique features, Gr FETs promise to be suitable for providing key insights into the dynamics of the electric potentials at the interfaces of biomolecular condensates. Here, we aimed to quantify the interfacial electric potentials and to determine the interaction mechanism by capitalizing on the unique features of graphene as an electrostatic charge transfer gauge and a well-described neuronal condensate of synapsin 1, a major synaptic protein implicated in neurotransmission.^20^ Our data show that synapsin condensates interact with the membrane interface in a charge-dependent manner. Using Gr FETs that allow measurements under physiological conditions, we gain insight into the remarkable ion potential at the interface of synapsin condensates that is completely absent in the single-phase regime of synapsin. Our results indicate EDL formation between synapsin and graphene. Thus, our findings strongly support that biomolecular condensates form electric field gradients at the interface to the membrane and that they could act as charge centers in the cell.

Synapsin 1 forms biomolecular condensates at physiological salt concentration and pH, due to the presence of its long, positively charged (pI 12.3), intrinsically disordered region (IDR).^21,22^ Synapsin condensates can sequester liposomes and native synaptic vesicles,^23^ presumably due to the specific protein and lipid interactions.^24^ To characterize the interaction of synapsin condensates with membranes, we coincubated giant unilamellar vesicles (GUVs) with condensates of synapsin 1. Synapsin condensates exhibited a characteristic interface wetting of membranes (Figure 1a,b). The synapsin molecules within condensates at the interface of GUVs remain mobile, as indicated by the recovery of fluorescence after photobleaching (Figure 1c). The increase in salt concentration altered the contact line between condensates and membranes, suggesting a charge-driven effect (Figure 1d,e). Even in a dilute, single phase synapsin 1 binds to lipid membranes.^25^ To determine whether the condensate wetting of membrane interfaces is independent of the N-terminal lipid binding, we coincubated condensates containing synapsin full length (FL) or synapsin IDR (a.a., 416–705) with GUVs. Indeed, synapsin IDR-mediated condensates also wetted the membrane interfaces, despite the absence of the high-affinity lipid-binding region within its N-terminus (Figure 1f,g).

**Figure 1:**
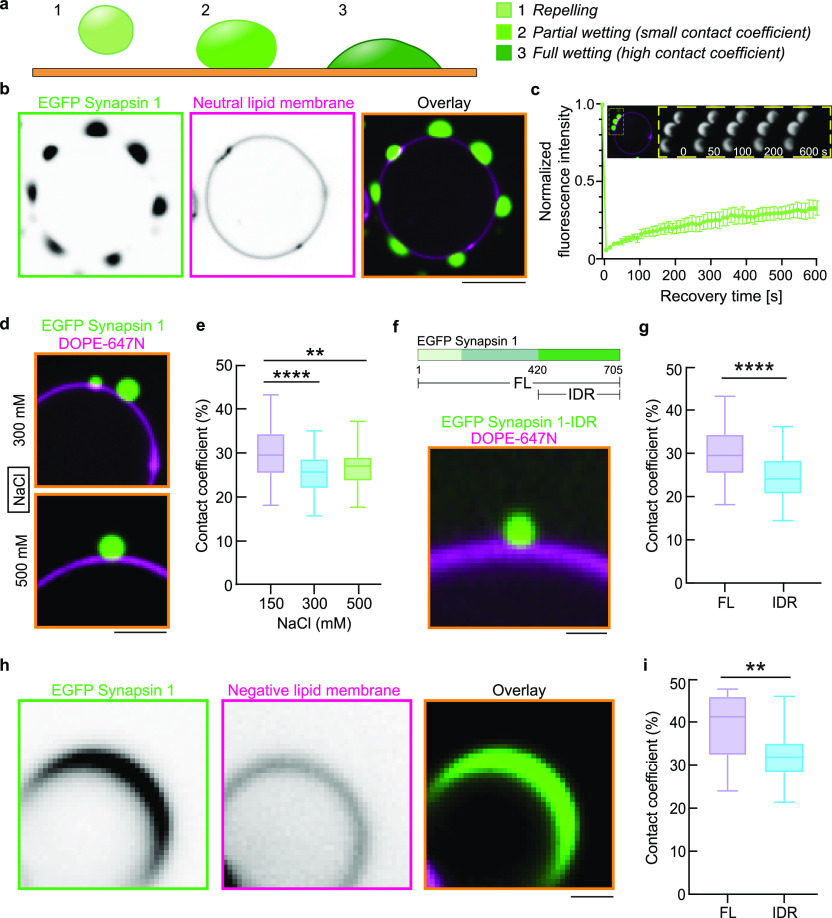
Synapsin condensates wet the interface of giant unilamellar vesicles (GUVs) in a charge-dependent manner. (a) Scheme of potential scenarios for the interaction between biomolecular condensates and membranes indicating repulsion of condensates from the membrane interface, partial- and full-wetting. (b) Synapsin condensates (5 μM EGFP-Synapsin 1 in 5% PEG 8,000) wet the interface of GUVs (99 mol % DOPC spiked with 1 mol % DOPE-647N for visualization) in the buffer with physiological salt concentration (i.e., 150 mM NaCl). Scale bar, 10 μm. (c) Fluorescence recovery after photobleaching suggests that synapsin 1 remains mobile within condensates at the interface of GUVs. Top: exemplary images of synapsin condensates before bleaching and upon recovery of fluorescence. Bottom: Normalized fluorescence intensity of synapsin condensates upon photobleaching represented as mean ± SEM. (d) Synapsin condensates (5 μM, EGFP-Synapsin 1 in 5% PEG 8,000) wet the interface of GUVs in the buffer 300 mM (top) and 500 mM (bottom) NaCl. Scale bar, 5 μm. (e) Quantification of the fraction of synapsin condensates in contact with the membrane interface shows that the contact drops with the increase in salt concentration. (f) Intrinsically disordered region of synapsin 1 (EGFP-Synapsin 1 IDR) is sufficient to form condensates that wet the interface of GUVs. Top: protein scheme, bottom: representative confocal image. Scale bar, 2 μm. (g) Quantification of the fraction of the circumference of EGFP-Synapsin 1 -FL or -IDR which is in contact with the neutrally charged membrane interface. (h) Representative image of EGFP-Synapsin 1 full length (FL). Negatively charged lipids (i.e., as in *a* supplemented with 25 mol % DOPS) at the interface of GUVs trigger the full wetting of synapsin condensates. Scale bar, 2 μm. (i) Quantification of the fraction of the circumference of EGFP-Synapsin 1 -FL or -IDR which is in contact with the negatively charged membrane interface. For *e*, *g*, and *h*, each data set is averaged from three reconstitutions and the error bars represent standard deviation.

Given the pronounced charge of synapsin 1, we aimed to explore whether the presence of negatively charged phospholipid headgroups in GUVs will affect the extent of synapsin condensate wetting. In fact, the wetting of synapsin condensates was significantly more pronounced when GUVs contained a negatively charged surface (Figure 1h,i). Together, these data strongly support the charge active interface of synapsin condensates, further prompting the investigation of how the electrical properties of synapsin change as it undergoes condensate formation.

As the increase in salt concentration or affecting the charge state of GUVs both play a role in the synapsin condensate wetting of the membranes, we set out to determine whether a shift from a one-phase to a condensed state of synapsin would impact the electrical properties at the interface. For this, we designed and employed graphene field-effect transistors (Gr FETs). An optical micrograph of one device is presented in Figure 2a. Graphene-based sensors have demonstrated capabilities to detect a single electron transfer event.^9^ Unlike spectroscopy-based techniques that indirectly measure electrical properties of the interface,^26^ Gr FETs have their entire volume exposed to the adsorbed species. Hence, they allow effective counting of the change in the number of free electrons as the interface to the adsorbate forms.^18,27,28^

**Figure 2:**
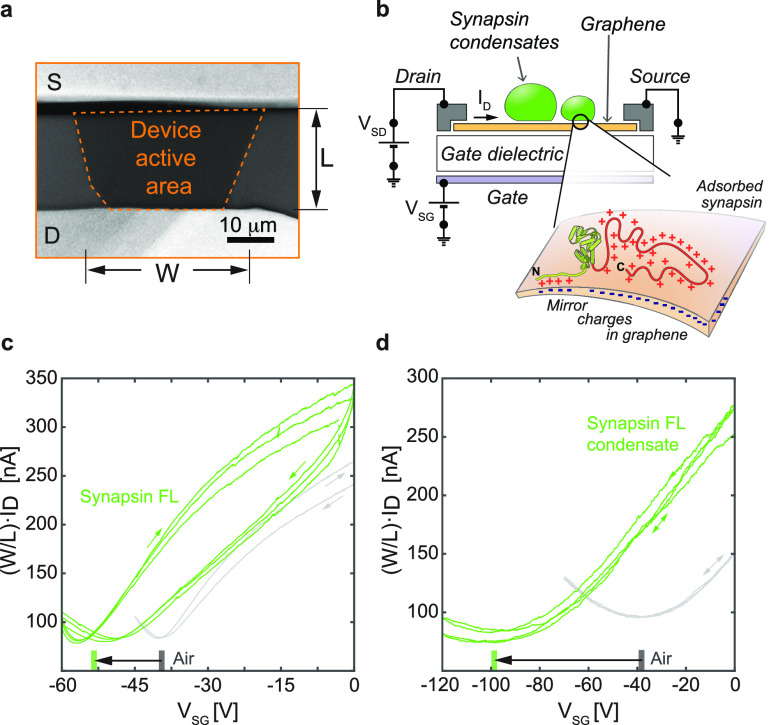
Electric field induced by synapsin 1 gauged with graphene sensors. (a) An optical micrograph of one device. The estimated region for the length (*L*) and width (*W*) of the active area of Gr FET is highlighted with dashed lines. (b) Scheme of the device cross-section indicating the applied biases (voltage) and the current flow direction. The magnification illustrates the electric double layer formation at the interface-to-analyte region. (c–d) Transfer characteristics (*I*_D_(*V*_SG_)) of Gr FETs right before exposure to the analyte (gray) and while in the solution (green), respectively for (c) synapsin full length (FL) and (d) condensates of synapsin full length. The bars on the *x*-axis in *c–d* indicate the mean position of the CNPs for each case, while the arrows indicate the shift of the transfer curves upon interaction with the analytes.

A scheme of the Gr FET connections and the applied biases is provided in Figure 2b. Note that biomolecules are not immobilized on the surface of the device by functional anchoring groups, but the binding mechanisms are naturally provided by the electrostatic interactions at the interface. Application of a small and constant bias (voltage) between the two electrodes (*V*_SD_) results in the measured current flow through graphene sheet, drain–source current (*I*_D_). The changes in *I*_D_ are related to the change in the electric field experienced by the graphene sheet. To quantify this relation, the dependence of the *I*_D_ upon cyclic sweeping of the source-gate bias (*V*_SG_), so-called electrical transfer curve, was measured in air moments before the analyte exposure (gray curves in Figure 2c–d; Figure S1). The amount of shift that the *I*_D_(*V*_SG_) curve experiences can be translated to the change in the electron concentration (Δ*e*^*–*^) in graphene.^29,30^ Synapsin 1 in the buffer solution shows shifting of the *I*_D_(*V*_SG_) curves to the lower *V*_SG_ values, indicating the accumulation of electrons in graphene as the interface to the analyte forms. Reference measurements of the buffer solution and pure deionized water are provided in the Supporting Information (Figure S1). Further, in all cases the slopes of the transfer curves remain mostly unaffected upon the exposure to the analytes. The major change is observed in shifting of the curves with respect to the applied *V*_SG_, indicating the electrostatic origin of the main interaction mechanism. Especially in the case of synapsin 1 FL condensates (Figure 2d) a strong shift of the apparent charge neutrality point of graphene (CNP – minima in the *I*_D_(*V*_SG_) curves) to the negative *V*_SG_ values was observed.^31^ As synapsin is positively charged, the data suggest that the observed change is a consequence of the EDL formation between graphene and the adsorbed synapsin, as illustrated in Figure 2b.

Next, we set out to determine whether there is a difference in the electrostatic properties of the interface when a solution containing a single phase or condensates of synapsin 1 is applied to Gr FETs. To trigger the condensation of synapsin, we add a crowding reagent (3% PEG 8,000 final concentration). The comparison of the transfer curves in air and in analyte solutions provides a strong indication that synapsin 1 interacts stronger with graphene in a condensate than in a dilute form (Figure 2c,d).

However, as both synapsin 1 and its condensate introduce the same direction of the CNP shift, a better comparison is achieved by following the kinetics of the response as the analytes are introduced and the interface forms. For this, we record the temporal evolution of *I*_D_ without an applied gate bias and introduce the analyte. The measured changes in the current, *i.e.*, the interaction kinetics at the interface, are expressed as the change in the electron concentration Δ*e*^–^(*t*) and normalized per unit area of the device’s active surface for comparison (Figure 3). An increase in the Δ*e*^–^(*t*) curves indicates the accumulation of electrons in graphene, which is also termed the n-type doping or rising of graphene’s Fermi level.

**Figure 3:**
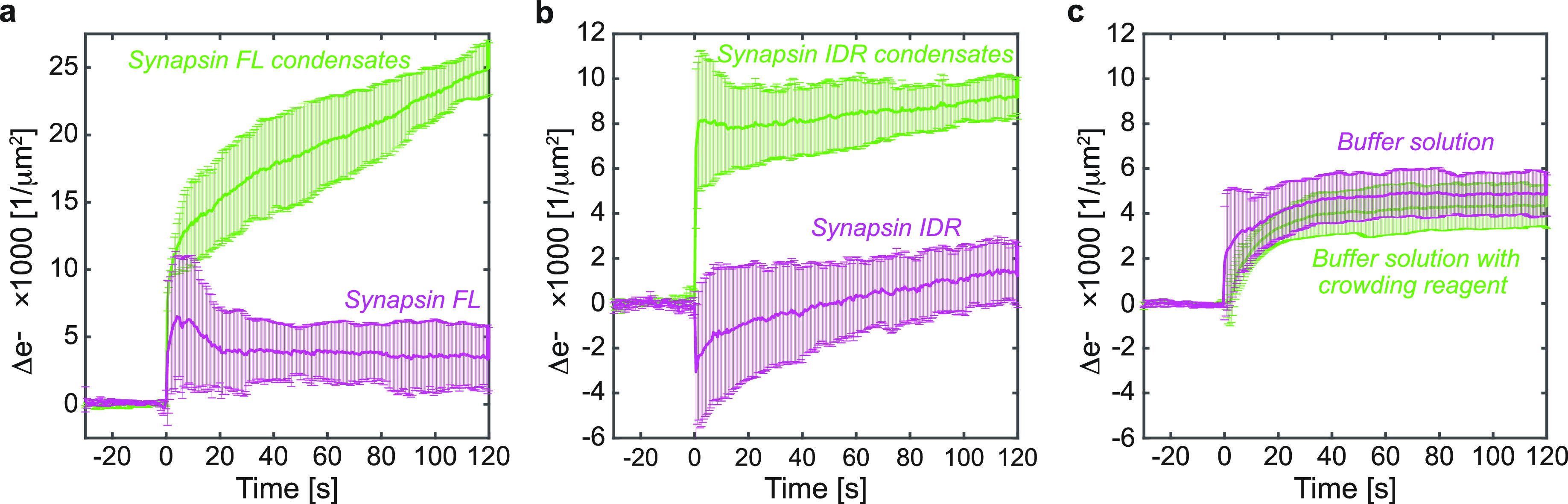
Kinetics of the interface formation for dilute and condensate states of synapsin 1. Measurements of the change in the electron concentration as a function of time Δ*e*^–^(*t*). The analyte droplets are introduced to the interface of the Gr FETs at 0 s. Each data set is averaged between three FETs and the error bars represent standard deviation. Measured currents are expressed as a relative charge in the charge concentration and are normalized to the active area of each device. (a) A comparison of synapsin full length (FL) in dilute and condensate states. (b) Same as in *a* only with synapsin intrinsically disordered region (IDR). (c) A reference experiment measuring the response of the devices exposed to a buffer solution without or with the crowding reagent. Of note, the scales in *b* and *c* are set the same for clearer comparison.

We observed a significant interfacial difference in the interaction between single-phase synapsin 1 and synapsin 1 condensates with Gr FET. In the condensate state of synapsin 1 FL, a continuous increase of the Δ*e*^–^(*t*) curves was observed (Figure 3a, green curves). In contrast, a single-phase synapsin FL gives only a minor increase in the rate of charging (Figure 3a, red curves) and saturates about 20 s after the exposure of the device to the analyte. Consistently with the observed wetting of GUVs, the IDR region of synapsin 1 was sufficient to recapitulate the trend in the Δ*e*^–^(*t*) between the single phase and condensate states (Figure 3b). This clearly indicates that indeed the IDR region is crucial for the formation of the EDL. As a reference, the sole exposure of the buffer solution without or with the crowding reagent showed an initial electron accumulation in graphene, followed by a rapid saturation (Figure 3c). This result indicates that the measurements for synapsin condensates, both FL and IDR, are independent of the crowding reagent. Especially when compared to the condensate state of synapsin full length, the buffer with the crowding reagent introduces over an order of magnitude smaller change in the accumulated charge per unit area.

It is important to note that the density transitions of synapsin 1 to form condensates imply that there will always be some number of synapsin 1 molecules in the dilute phase as well. While in our current iteration of the measurement setup it was not possible to precisely quantify the amount of the unsegregated proteins, the quantitative comparison between the Δ*e*^–^(*t*) curves for the dense and the dilute phases in Figure 3a,b remains undetermined. Despite this limitation, the Gr FET measurements show a clear trend and confirm that the condensate states of proteins profoundly change the electrical properties of the interfaces that they form.

To further support the formation of the EDL and not a direct charge transfer between the adsorbate and graphene, we performed the experiments with an atomically thin dielectric separator placed between graphene and the adsorbates. For this, we have used exfoliated hexagonal boron nitride (hBN) flakes with a thickness (*d*) ranging from 3 to 40 nm. A scheme of the experiment is presented in Figure 4a. If the observed change in the graphene electrical response is generated by a direct exchange of the electrons with the adsorbate, then already at 3 nm thickness of a dielectric separator, the energy barrier for the exchange would be sufficiently high to prohibit the electrical response. However, if the electrical response of Gr FETs is a consequence of the EDL formation, then the response should prevail and the experienced electric potential by graphene should decay as 1/*d* (*d*, dielectric thickness).

**Figure 4:**
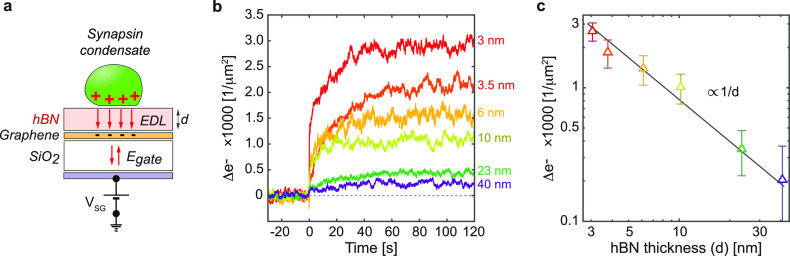
Probing the electric double layer (EDL) formation for synapsin 1 condensates. (a) A scheme of the layered structure involving hexagonal boron nitride (hBN) as an atomically thin dielectric separator between synapsin 1 condensates and their mirror charges in graphene. Red arrows indicate the electric fields introduced by the synapsin condensates (top, hBN region) and by the gate electrode (bottom, gate dielectric SiO_2_). (b) Measurements of the change in the electron concentration as a function of time Δ*e*^–^(*t*), with varying hBN layer thickness between 3 and 40 nm, as indicated on the right side of the graph. (c) Double logarithmic plot of the saturation Δ*e*^–^(*t*) values as the function of *d*. Solid line represent 1/*d* fit (*d*, dielectric thickness).

Figure 4b shows Δ*e*^–^(*t*) curves for the experiments with synapsin 1 FL with a varied dielectric separation layer thickness. Evidently, the response has a trend similar to that of the experiments carried out without the separation layer (Figure 3a), with two differences: Saturation is reached sooner and the intensity of the change reduces with an increase of the dielectric layer thickness. To evaluate the dependence of the Δ*e^–^* on the dielectric thickness, we plot the saturation values from Figure 4b (mean values of the last 20 s and their corresponding standard deviations) as a function of the hBN thickness. The results are provided in Figure 4c on a double logarithmic scale. Here, a linear fit was carried out, confirming the *1/d* dependence of the response, that is, the formation of EDL.

It is important to note that in comparison to the cellular membrane, graphene and hBN possess different physicochemical properties. Consequently, the precise interaction and binding mode of synapsin 1 could differ on graphene and hBN from their behavior on cellular membranes. However, we observe identical trends for both synapsin 1 full-length and synapsin 1 IDR condensates when comparing the wetting experiments on neutral and charged GUVs, with the electrical response of Gr-FETs, highlighting the functional link between the EDL at the interface and the wetting capacity of synapsin condensates on membranes.

There are three main implications of these data. First, the data clearly indicate that the electrical environment at the interface to the surroundings of the same protein is substantially different when the protein is in a single phase versus within a condensate. Second, the experiments strongly support the idea that the formation of EDL occurs between synapsin condensates and graphene, indicating that the condensation drives the formation of the electric potential gradient at the interface of biomolecular condensates. Finally, these experiments suggest that synapsin/synaptic vesicle condensates could act as a charge center at the synaptic boutons, which provides a new layer for the regulation of neurotransmission.

## Supporting Information Available

Additional experimental details, materials, and methods, including the supplementary figure of reference electrical transfer curves. (PDF)

## Abbreviations

EDL: electric double layer
Gr FET: graphene field effect transistors
GUV: giant unilamellar vesicle
FL: full length
IDR: intrinsically disordered region
CNP: charge neutrality point
DI water: deionized water
PEG: polyethylene glycol
DOPC: 1,2-dioleoyl-*sn*-glycero-3-phosphocholine
DOPS: 1,2-dioleoyl-*sn*-glycero-3-phospho-l-serine
DOPE: 1,2-dioleoyl-*sn*-glycero-3-phosphoethanolamine
hBN: hexagonal boron nitride.
