# Supplementary material for: Electric Potential at the Interface of Membraneless Organelles Gauged by Graphene
Source: Nano Lett. 2023 Oct 20;23(23):10796–801. doi: 10.1021/acs.nanolett.3c02915 (PMC10722609; doi:10.1021/acs.nanolett.3c02915)
Supplement: Supplementary file 1 — nl3c02915_si_001.pdf [file nl3c02915_si_001.pdf]

***Supporting information for:***

# Electric Potential at the Interface of Membraneless Organelles Gauged by Graphene

*Christian Hoffmann[1,‡], Gennadiy Murastov[2, ‡], Johannes Vincent Tromm[1,‡], Jean-Baptiste Moog[1], Muhammad Awais Aslam[2], Aleksandar Matkovic[2,*] & Dragomir Milovanovic[1,*]*

[1]Laboratory of Molecular Neuroscience, German Center for Neurodegenerative Diseases (DZNE), 10117 Berlin, Germany.
[2]Chair of Physics, Department Physics, Mechanics and Electrical Engineering, Montanuniversität Leoben, 8700 Leoben, Austria
[‡]These authors contributed equally to this work
[*]Correspondence should be addressed to:
aleksandar.matkovic@unileoben.ac.at and dragomir.milovanovic@dzne.de

***Supporting information file includes:***

- Methods
- Supplementary Figure 1
- References that pertain to the Methods section

*Methods*

**Protein Purification.** The recombinant proteins, full length (FL) and the intrinsically disordered region (IDR, a.a. 416-705) of EGFP-Synapsin 1, were expressed in mammalian expression system and purified as previously described[1,2].

**Giant Unilamellar Vesicles (GUVs).** Lipids (Avanti polar lipids, Inc.) used for Giant Unilamellar Vesicles (GUVs) were dissolved in Chloroform and stored in glass vials at -20°C respectively. The following lipid species were used: (i) 18:1(Δ9-Cis)PC / 1,2-dioleoyl-sn-glycero-3-phosphocholine (DOPC), (ii) 18:1 PS / 1,2-dioleoyl-sn-glycero-3-phospho-l-serine (DOPS), (iii) Atto647N coupled to 1,2-dioleoyl-sn-glycero-3-phosphoethanolamine (DOPE-Atto647N), a kind gift from Dr. Vladimir Below, Max Planck Institute for Multidisciplinary Natural Sciences, Göttingen, DE. The different lipid components were mixed at a molar ratio of 3:1 for the DOPC:DOPS condition (i.e., 3.75 mM DOPC and 1.25 mM DOPS), while the DOPC condition consisted of 5 mM DOPC. In both conditions, the lipid solutions were spiked with the fluorescently labeled lipid DOPE-Atto647N.

The GUVs were generated by electroformation using Vesicle Prep Pro and respective indium tin oxide (ITO)-coated glass slides[3,4] Specifically, the coated glasses were cleaned with Milli Q water, 70% Ethanol (VWR Chemicals) and ≥99.5% Isopropanol (Carl Roth GmbH). The ITO-coated side of the glass was identified using a voltmeter, and 20 µl of the lipid solution was pipetted on the conducting surface. The solution was left at RT to let the solvent of the lipids evaporate. After 20 min, silicon grease was applied to the 16 mm O-ring (Nanion Technologies GmbH) was was then placed around the dry lipid film. 250 µl of an aqueous solution containing 500 mM D-Sorbitol (MP Biomedicals) was pipetted inside the O-ring and a second ITO-covered

glass slide was inserted on top with the conducting side facing downwards. A protocol was run on the Vesicle Prep Pro (A = 3 V; f = 5 Hz; T = 36°C; $t_{main}$ = 2 h; $t_{rise}$ = 5 min; $t_{fall}$ = 5 min). During the rise and fall period, a linear increase (rise) and decrease (fall) of the amplitude with a constant frequency and temperature was carried out respectively. The GUVs were obtained with a cut-off pipette tip by pulling the solution up and pushing it down gently several times. Then, the vesicle solution was placed in a reaction tube and stored at 4°C until the start of the experiments.

For the reconstitutions, the condensates were formed as described in[2]. In short, the protein was incubated with Polyethylenglycol 8,000 (PEG 8,000) for 15 min and the mixture was then pipetted on top of the GUVs on a glass bottom microscopy dish. The final composition of the sample solution consisted of 5 µM protein-of-intertest, 5% PEG 8,000, 18.75 mM Tris-HCl (pH = 7.4), 0.375 mM TCEP and 125 mM D-Sorbitol. The concentration of NaCl was 150 mM unless otherwise indicated. In order to minimize evaporation, Milli Q water was placed at the periphery of the microscopy dish before adding any reaction component on the surface.

**Microscopy and image analysis.** The images were acquired using the Eclipse Ti Nikon Spinning Disk Confocal CSU-X, equipped with 2 EM-CCD cameras (AndorR iXon 888-U3 ultra EM-CCD), Andor Revolution SD System (CSU-X), objectives PL APO 60/1.4NA oil immersion lens. Excitation wavelengths were: 488-nm for EGFP-Synapsin 1 (both IDR and FL); 638-nm for DOPE-647N. The laser intensities were 0.013 µW for EGFP-Synapsin 1-FL and 0.086 µW for EGFP-Synapsin 1-IDR. The laser intensities at 638 nm had to be varied depending on the selected GUV but were not higher than 1.520 µW. The exposure time was 200 ms for all experiments. EM Gain (30 MHz at 16-bit) Multiplier was set to 300 and Piezo stage z-motor was used to collect z-series.

For fluorescence recovery after photobleaching (FRAP) assays, the bleach ROI was set to 1.12 µm of diameter and bleached at the maximum transmission intensity (488 nm = 4.240 µW and 638 nm = 7.820 µW) in 15 loops. During the experiments, six images were acquired before bleaching in one-second intervals, followed by the bleaching, and another acquisition phase during which one image was acquired every second over ten minutes. The reference ROI was assigned to a condensate, not in contact with any GUV. The microscopy data was acquired using the software NIS Elements 5.21.02 and then analyzed with Fiji ImageJ (NIH).

For the analysis of the FRAP data, the mean fluorescent intensity inside the bleached area was measured every second over the course of the entire recording. A correction factor was calculated based on the reduction of fluorescence outside of the bleached area; the corrected values were then normalized according to the minimum and maximum values over the total span of the recording. The contact coefficient was quantified in the following manner: A circumference of the condensate (c) was marked as well as at the contact interface between the condensate and the GUV (l). The contact coefficient represents the ratio l/c, expressed as a percentage. For all contact coefficients, at least five different condensates from three independent reconstitutions were quantified. For statistical analysis, unpaired, two-tailed t-tests were performed. Two asterisks (**) indicate a p-value $< 0.01$; four asterisks (****) indicate a p-value $< 0.0001$. All the plots were generated in GraphPad PRISM 9.

**Graphene Field Effect Transistors (Gr FET).** Gr FETs were made on highly doped n type Si wafers with 300 nm wet thermal oxide layer. Monolayer flakes were isolated via micromechanical exfoliation procedure[5] from natural graphenium flakes (from NGS) by multiple cleavage with sticky tapes (Nitto Denko ELP BT150ECM), and subsequently deposited on the Si/$SiO_2$ chips. Thin flakes were pre selected by optical microscopy based on established monolayer contrast[6].

The monolayer thickness was confirmed by Raman spectroscopy[7], using Horiba LabRam HR Evolution confocal spectrometer with 1800 lines/mm gratings, 532 nm laser source, and excitation power of 3.2 mW.

Electrodes were deposited by deterministic dry transfer of graphite flakes[8] using poly(dimethylsiloxane) (PDMS) stamps. Electrode flakes were prepared by cleaving of "needle" graphite crystals (from NGS), and transferred to PDMS stamps for selection and alignment with graphene on $SiO_2$/Si support. The same stamping procedure was employed with hBN layers that were used for capping of the devices in the experiments with the dielectric separator layers. Stacking of the heterostructures was carried out in a custom-built ambient transfer stage setup. By avoiding conventional photolithography or electron beam lithography, pristine surface of the channel is preserved[9]. Such process ensures that the adsorption events are not governed by contaminants or resist residues in the channel active area. Size of the device active area was estimated from optical micrographs and cross checked by atomic force microscopy measurements. All employed devices had a two terminal and global $SiO_2$ back gate geometries. Typical channel lengths and widths were between 10 μm and 20 μm.

**Electrical measurements.** Two types of the electrical measurements were carried out: electrical transfer characteristic measurements (drain current as a function of the global gate bias sweeping), and sensing the charge transfer. In all cases, Keithley 2636A Source-Meter attached to the Instec probe station was used. During both type of electrical measurements, the bias (voltage) between the source and the drain electrodes was kept at 10 mV, yielding the drain currents in a range of 100–1000 nA.

For the measurements of the transfer characteristics cyclic sweeping of the back gate bias with minimum of three repeated cycles was done in air immediately before the sensing experiments, with sweep rates of 0.5 V/s. When possible, the same process was repeated in liquid after the drain current saturates (about 120 s to 180 s, with an exception of the synapsin 1 FL solution where longer times are needed to achieve saturation). In liquid a minor increase in the forward and backward sweeping hysteresis was observed, which could be attributed to the interaction with water molecules. If exposed to pure deionized water, the hysteresis was observed to be emphasized.

For the sensing of the charge transfer, the gate bias was set to 0 V, while 10 mV of the source drain bias was applied. The current through the device was measured in intervals of 20 ms. After 30-60 s of stable current level in air, a 2 μL droplet of the solution was drop casted covering the device active area and the electrodes, but not the edges of the chips to ensure that there is no interference with the grounded back gate electrode. Formation of a parallel conductive path through the solution can be neglected as the overall current values do not change significantly, that is, the predominant change of the $I_D(V_{SG})$ curves upon exposure to the analytes is the shift of the curves with respect to analyte-specific $V_{SG}$ offset. This is also obvious from the fact that the minimal value of the charge neutrality point has only a minor change when comparing the transfer characteristics measured in air and in the liquids.

To convert from the total current measured across the device to the $\Delta e^-(t)$ per unit area, transconductance curves measured before the exposure to liquid were used to estimate the gate bias equivalent change by interaction with the solution. Afterwards, considering the active area of the devices (area of the exposed graphene monolayers) and known back gate capacitance it is

possible to express the detected change in the current as the number of charges that change in the graphene layer upon the interaction with the liquid.

For each data set presented in Figure 3, three Gr FETs were used, starting from initially both n and p type unintentionally doped devices. As a result, for the same liquid the opposing initial values (in the n- or p- branches) of the Fermi level would introduce reduction or increase in the measured drain current. However, when expressed as $\Delta e^-(t)$, the response of both initially n and p type devices was found to be consistent. The initial variations of the Fermi level were estimated to be within the range of ±100 meV.

For n-doping, if the device is becoming n-doped this indicates that surely the number of the free electrons in graphene is increasing. Consequently, graphene layer becomes negatively charged. This implies that the formation of the interface drives an increase of the Fermi level in graphene, shifting it to a higher value. Consequently, the $I_D(V_{SG})$ curves appear shifted to the left side with respect to the measurements before the analyte exposure. This amount of shift is measured in the applied gate voltage, and can be then expressed either as the shift of the Fermi level, or a change in the electron concentration. The opposite would occur for p-doping. Here, electrons are leaving graphene. One could also describe this as that holes are accumulating in graphene. Consequently, the Fermi level lowers i.e., the graphene layer becomes positively charged.

Measuring the change in the electrical properties of graphene upon analyte exposure pinpoints what change this has introduced in the electron gas of graphene. However, the observed fluctuations of the free carriers upon the interface formation could be originating from two scenarios: In the first scenario, a charged adsorbate from the analyte solution comes in contact

with graphene and physically exchanges electrons across the graphene/adsorbate interface. In the second scenario, the adsorbate comes in contact with graphene, but cannot exchange the charge. This leads to graphene experiencing the electrostatic field of the adsorbate and consequently the needed access charge is provided by the electrical circuit i.e., the electrons never cross the graphene/adsorbate interface.

**SUPPLEMENTARY FIGURE**

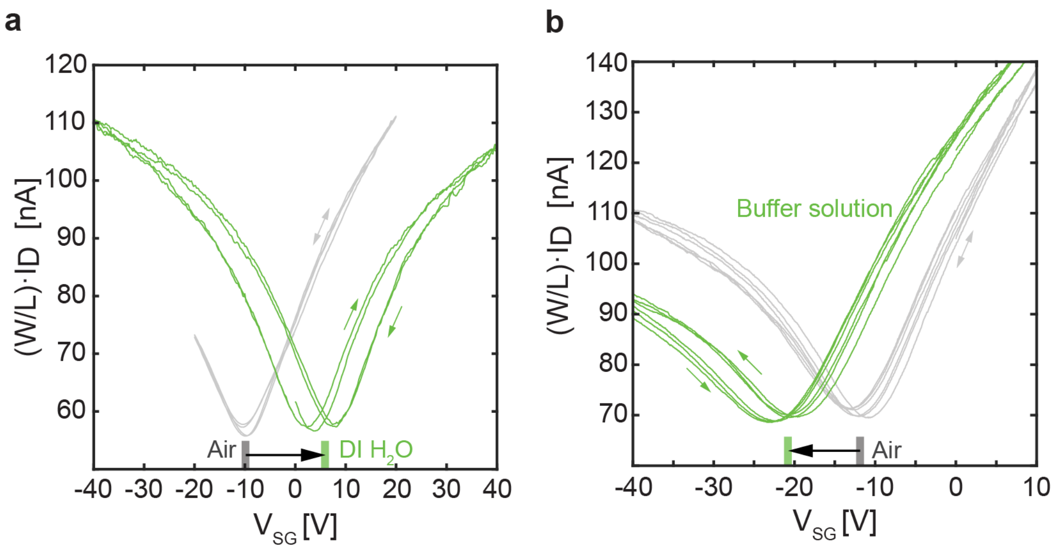


**Figure S1: Reference electrical transfer curves.** Transfer characteristics ($I_D(V_{SG})$) of Gr FETs right before exposure to the analyte (gray) and while in the solution (green) for **(a)** pure deionized (DI) $H_2O$, **(b)** buffer solution. In the case of DI water, a shift of the characteristics in the positive $V_{GS}$ direction and pronounced hysteresis were observed. Both the observed $\Delta e^-$ and the hysteretic device behavior are attributed to the interaction of water molecules with graphene and the water dipole response to the sweeping of the gate fields[10,11].

## REFERENCES


(1) Milovanovic, D.; Wu, Y.; Bian, X.; Camilli, P. D. A Liquid Phase of Synapsin and Lipid Vesicles. *Science* **2018**, *361* (6402), 604 607. https://doi.org/10.1126/science.aat5671.

(2) Hoffmann, C.; Sansevrino, R.; Morabito, G.; Logan, C.; Vabulas, R. M.; Ulusoy, A.; Ganzella, M.; Milovanovic, D. Synapsin Condensates Recruit Alpha-Synuclein. *J Mol Biol* **2021**, 166961. https://doi.org/10.1016/j.jmb.2021.166961.

(3) Kreir, M.; Farre, C.; Beckler, M.; George, M.; Fertig, N. Rapid Screening of Membrane Protein Activity: Electrophysiological Analysis of OmpF Reconstituted in Proteoliposomes. *Lab Chip* **2008**, *8* (4), 587–595. https://doi.org/10.1039/b713982a.

(4) Ramadurai, S.; Holt, A.; Krasnikov, V.; Bogaart, G. van den; Killian, J. A.; Poolman, B. Lateral Diffusion of Membrane Proteins. *J Am Chem Soc* **2009**, *131* (35), 12650 12656. https://doi.org/10.1021/ja902853g.

(5) Prinz, J.; Matković, A.; Pešić, J.; Gajić, R.; Bald, I. Hybrid Structures for Surface-Enhanced Raman Scattering: DNA Origami/Gold Nanoparticle Dimer/Graphene. *Small* **2016**, *12* (39), 5458–5467. https://doi.org/10.1002/smll.201601908.

(6) Blake, P.; Hill, E. W.; Neto, A. H. C.; Novoselov, K. S.; Jiang, D.; Yang, R.; Booth, T. J.; Geim, A. K. Making Graphene Visible. *Appl Phys Lett* **2007**, *91* (6), 063124. https://doi.org/10.1063/1.2768624.

(7) Malard, L. M.; Pimenta, M. A.; Dresselhaus, G.; Dresselhaus, M. S. Raman Spectroscopy in Graphene. *Phys Reports* **2009**, *473* (5–6), 51–87. https://doi.org/10.1016/j.physrep.2009.02.003.

(8) Castellanos-Gomez, A.; Buscema, M.; Molenaar, R.; Singh, V.; Janssen, L.; Zant, H. S. J. van der; Steele, G. A. Deterministic Transfer of Two-Dimensional Materials by All-Dry Viscoelastic Stamping. *2D Mater* **2014**, *1* (1), 011002. https://doi.org/10.1088/2053-1583/1/1/011002.

(9) Aslam, M. A.; Tran, T. H.; Supina, A.; Siri, O.; Meunier, V.; Watanabe, K.; Taniguchi, T.; Kralj, M.; Teichert, C.; Sheremet, E.; Rodriguez, R. D.; Matković, A. Single-Crystalline Nanoribbon Network Field Effect Transistors from Arbitrary Two-Dimensional Materials. *Npj 2D Mater Appl* **2022**, *6* (1), 76. https://doi.org/10.1038/s41699-022-00356-y.

(10) Levesque, P. L.; Sabri, S. S.; Aguirre, C. M.; Guillemette, J.; Siaj, M.; Desjardins, P.; Szkopek, T.; Martel, R. Probing Charge Transfer at Surfaces Using Graphene Transistors. *Nano Lett* **2010**, *11* (1), 132–137. https://doi.org/10.1021/nl103015w.

(11) Bagheri, M. H.; Loibl, R. T.; Schiffres, S. N. Control of Water Adsorption via Electrically Doped Graphene: Effect of Fermi Level on Uptake and H2O Orientation. *Adv Mater Interfaces* **2021**, *8* (18), 2100445. https://doi.org/10.1002/admi.202100445.
